# Repetitive high-sustained gravitoinertial stress does not modulate pressure responsiveness to peripheral sympathetic stimulation

**DOI:** 10.1007/s00421-023-05354-6

**Published:** 2023-11-22

**Authors:** Michail E. Keramidas, Roger Kölegård, Antonis Elia, Håkan Sköldefors, Ola Eiken

**Affiliations:** 1 Division of Environmental Physiology, Swedish Aerospace Physiology Center, KTH Royal Institute of Technology, Berzelius väg 13, Solna, 171 65 Stockholm, Sweden; 2 Swedish Air Force, Uppsala, Sweden

**Keywords:** Arterial pressure, Cardiovascular reflex, Cold pressor test, G training, Hypergravity

## Abstract

**Purpose:**

We evaluated the hypothesis that repetitive gravitoinertial stress would augment the arterial-pressure response to peripheral sympathetic stimulation.

**Methods:**

Before and after a 5-weeks G-training regimen conducted in a human-use centrifuge, twenty healthy men performed a hand cold-pressor test, and nine of them also a foot cold-pressor test (4 min; 4 °C water). Arterial pressures and total peripheral resistance were monitored.

**Results:**

The cold-induced elevation (*P* ≤ 0.002) in arterial pressures and total peripheral resistance did not vary between testing periods, either in the hand [mean arterial pressure: Before =  + 16% vs. After =  + 17% and total peripheral resistance: Before =  + 13% vs. After =  + 15%], or in the foot [mean arterial pressure: Before =  + 19% vs. After =  + 21% and total peripheral resistance: Before =  + 16% vs. After =  + 16%] cold-pressor tests (*P* > 0.05).

**Conclusion:**

Present results demonstrate that 5 weeks of prolonged iterative exposure to hypergravity does not alter the responsiveness of sympathetically mediated circulatory reflexes.

## Introduction

Fighter pilots flying high-performance aircraft are commonly exposed to high-sustained gravitoinertial force field in the head-to-seat direction (i.e., + Gz; henceforth G denotes + Gz), eliciting exaggerated hydrostatic pressure gradients in the vasculature, with markedly elevated intravascular pressures in the dependent vessels, and reduced pressures in the vessels above the heart. The capacity to, in a relaxed state (i.e., without the use of anti-G strategies/garments), withstand enhanced G loads (relaxed G tolerance) is, hence, determined by the arterial-pressure responsiveness, preserving adequate ocular and cerebral perfusion (Balldin 1986; Green 2016; Pollock et al. 2021). G tolerance, which describes large inter-individual variability, appears to be influenced by several anatomical and functional features, mainly including the basal levels of arterial pressure and the heart-to-head vertical distance (Klein et al. 1969), the wall stiffness of the lower-limb precapillary resistance vessels (Eiken et al. 2012, 2022 ), and, during gradual/slow increments of the G load, also the function of sympathetic circulatory reflexes (Newman et al. 1998; Sundblad et al. 2016; Convertino 2001; Scott et al. 2013). Thus, in regards to the latter, a cross-sectional study in a cohort of non-pilots, has indicated that, compared to individuals with low gradual onset G tolerance (< 4.2 G), those possessing high G tolerance (≥ 5.5 G) exhibit an augmented pressure response to an acute sympathoexcitatory stimulus, namely the hand cold-pressor test (Sundblad et al. 2014). These inherent differences in arterial-pressure regulation were attributable to between-group variations in vasoconstrictor sensitivity, presumably associated with higher myogenic responsiveness in the high-G-tolerant individuals, rather than to changes in sympathetic outflow.

Recently, we demonstrated that 5 weeks of repeated + G exposures (G training) in a relaxed state, improved G tolerance, especially during rapid onset-rate elevation of the G load (Eiken et al. 2022). Such a response was ascribed predominantly to local adaptations, described by the reduced pressure distensibility of leg arteries/arterioles, elicited by the recurrent transmural pressure increases. Still, whether the long-term iterative hypergravity exposures might also have modulated sympathetically mediated cardiovascular reflex responses, contributing, at least partly, to the enhanced G tolerance, remains unknown.

Accordingly, the present study tested the hypothesis that repetitive gravitoinertial stress would augment the arterial-pressure response to peripheral sympathetic stimulation. To this end, we employed a within-subject design, wherein systemic hemodynamic responses were monitored during a hand cold-pressor test, before and after a 5-weeks G-training regimen performed in a human-use centrifuge. On the basis of previous evidence (Sundblad et al. 2014), we hypothesized that iterative increments in total peripheral blood-flow resistance (TPR) induced during the G training, might amplify the cold-induced arterial-pressure elevation, due to a more pronounced increase in TPR. In view of our finding that the vasoadaptations evoked by the G training were limited to the lower-limb vasculature (Eiken et al. 2022), a foot cold-pressor test was also conducted by a subset of subjects, at the same time points.

## Methods

This study is part of two larger projects, which were conducted between 2016 and 2022 in the experimental facilities of the Division of Environmental Physiology (Solna, Sweden), examining the effects of prolonged repeated hypergravity exposures on the human cardiovascular system (see Eiken et al. 2022; Keramidas et al. 2023). The study was approved by the Human Ethics Committee of Stockholm (Ref. no.: 2016/1889-31/4 and 2019/06542) and conformed to the Declaration of Helsinki. Prior to participation, written informed consent was obtained from all subjects.

### Subjects

Twenty healthy male flight-cadets of the Swedish Air Force volunteered to participate [mean (range) age: 24 (21–27) years, weight: 80 (70–94) kg, height: 180 (171–186) cm]; they were recruited to the study before attending any flight training. All subjects performed the hand cold-pressor test, and nine of them also conducted the foot cold-pressor test. An a priori power analysis was not performed for these specific datasets, because the current work dealt with a secondary question within the larger projects (see Eiken et al. 2022).

### Experimental protocol and measurements

Subjects underwent, in a 7.25-m radius human-use centrifuge (ASEA, Sweden), a 5-weeks G-training regimen, comprising three 40-min sessions per week. Subjects were seated upright in the tangentially pivoted centrifuge gondola, wherein the seat back reclines 28° from the vertical. They remained relax throughout each G exposure; they were thus not allowed to perform anti-G straining maneuvers (i.e., Valsalva, isometric contraction/tensing of skeletal muscles), did not wear anti-G suits, and were not exposed to positive pressure breathing. During each session, the G load was oscillated, at 1-min intervals and at 0.5 G/sec transition rate, between idle speed (1.4 G) and a G load corresponding to ~ 85% of the individual, rapid onset-rate G tolerance: the mean (range) G load was 2.8 (2.6–3.4) G in the 1st week, 3.0 (2.6–3.6) G in the 2nd week, 3.1 (2.6–3.6) G in the 3rd week, 3.2 (2.8–4.0) G in the 4th week, and 3.2 (2.9–4.1) G in the 5th week.

Before and after the 5-weeks G-training regimen, all subjects performed a hand cold-pressor test, and nine of them also a foot cold-pressor test. Both tests were performed with the subjects in an upright sitting position. Each test commenced with a 10-min baseline phase. Thereafter, subjects immersed their right hand or foot for 4 min in 4 °C water; they were instructed to remain relax, breathe normally, and avoid any Valsalva-like maneuver throughout. The hand and foot cold-pressor tests were separated by a ~ 15-min interval; their order, which remained constant in the two testing periods, was alternated among subjects: four and five of them performed first the hand and foot cold-pressor test, respectively. For the individual subject, the time of the day that the tests were conducted were the same in the two testing periods. The temperature in the laboratory was maintained at ~ 24 °C. Beat-to-beat systolic (SAP), diastolic (DAP) and mean (MAP) arterial pressures were measured continuously using a volume-clamp technique (Finometer, Finapres Medical Systems BV, Amsterdam, the Netherlands). The pressure cuff was placed around the middle phalanx of the third finger of the left hand, and the reference pressure transducer was positioned at the level of the heart. Before each test, a brachial cuff was attached on the same arm, and the calibration process was performed according to the manufacturer’s instructions. Heart rate (HR) was derived from the arterial-pressure curves as the inverse of the inter-beat interval. Cardiac stroke volume was estimated by a three-element model of arterial input impedance from the arterial-pressure waveform (Modelflow, Finometer; Wesseling et al. 1993). Cardiac output (CO) was estimated by multiplying HR by stroke volume, and TPR was calculated by dividing MAP by CO. Subjects were asked, every minute, to provide ratings of the immersed-limb pain (from 0—no pain to 10—maximal pain).

### Data and statistical analyses

Baseline values were calculated as the average of the final 5 min of the 10-min baseline phase. Data from the cold-water immersion phase were calculated as the average of the entire 4-min period. Data are presented as absolute values, and as relative (%) changes to baseline. Normality of distribution for all datasets was assessed with the D’Agostino–Pearson test. All data were analyzed with two-way [test phase (baseline × cold stress) × testing period (before × after G training)] repeated-measures analysis of variance (ANOVA). Sphericity was assessed using Mauchly’s test, and the Greenhouse–Geiser *ɛ* correction was applied when necessary. When ANOVA revealed a significant *F* value, the Bonferroni correction was used to adjust for multiple post hoc comparisons. Differences in pain perception and the relative changes in cardiovascular responses to cold were assessed with a Wilcoxon test and a paired two-tailed *t* test, respectively. Statistical analyses were conducted using Prism 10.0 (GraphPad Software Inc., San Diego, CA, USA). Unless otherwise stated, data are presented as mean values with standard deviation. The *α* level of significance was set a priori at 0.05.

## Results

**Fig. 1:**
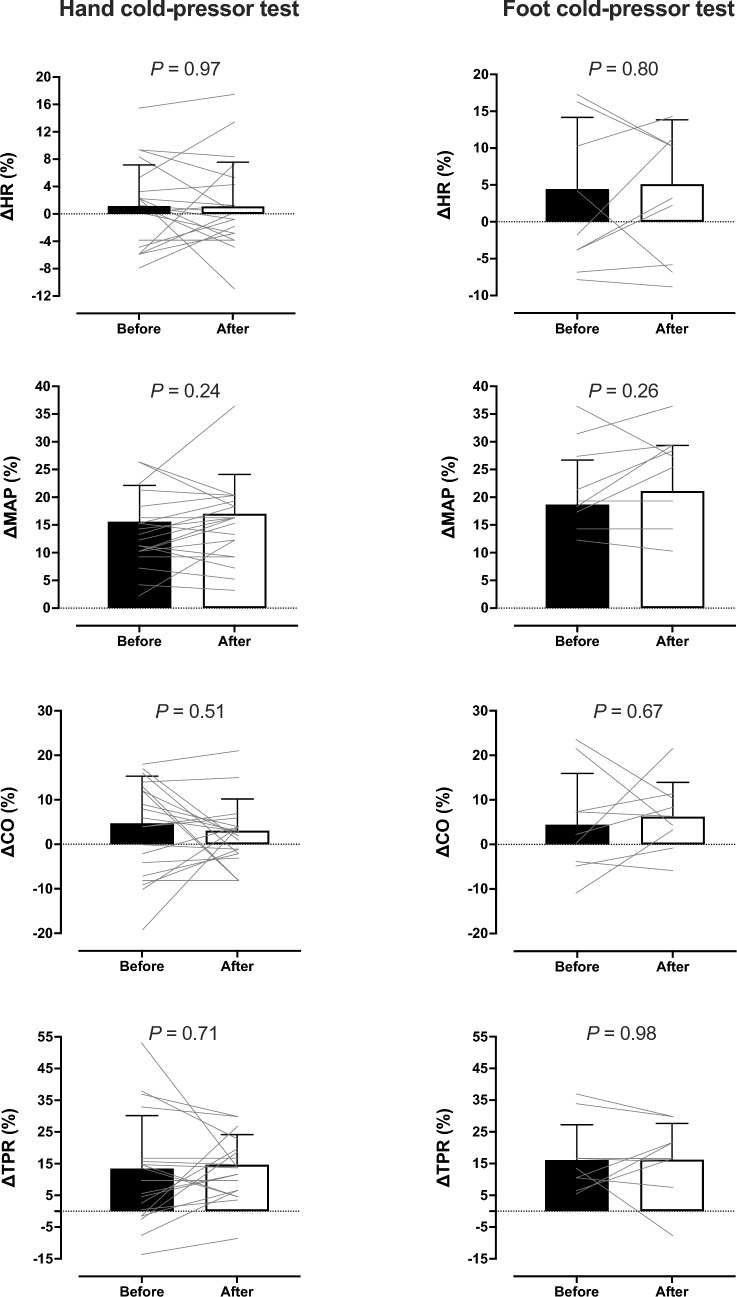
Mean (standard deviation) and individual values of changes relative to baseline in heart rate (HR), mean arterial pressure (MAP), cardiac output (CO) and total peripheral resistance (TPR) obtained during the hand (*n* = 20 men) and foot (*n* = 9 men) cold-pressor tests (4 °C water for 4 min), performed before and after a 5-weeks G training regimen. Data were analyzed with paired, two-tailed *t* test (*P* > 0.05)

Baseline cardiovascular values were similar across the tests (*P* > 0.05; Table 1). For either limb, the cold stress enhanced SAP, DAP, MAP and TPR (*P* ≤ 0.002); the increase did not vary between testing periods (Table 1 and Fig. 1). Neither the cold stress nor the G training altered HR and CO (*P* > 0.05; Table 1 and Fig. 1). The G training attenuated the pain sensation during the hand [mean (range): Before = 4.4 (3–7), After = 3.7 (2–5); *P* = 0.002], but not the foot [mean (range): Before = 4.8 (2–8), After = 4.1 (3–7); *P* = 0.13], cold-pressor tests.

**Table 1:** Cardiovascular values obtained during the hand and foot cold-pressor tests, performed before and after a 5-weeks G-training regimen

	Before G training		After G training		*P* value		
	Baseline	Cold stress	Baseline	Cold stress	Test phase	Testing period	Interaction
Hand cold-pressor test (*n* = 20 men)							
HR (beats/min)	71 (13)	72 (13)	68 (12)	68 (12)	0.45	0.11	> 0.99
MAP (mmHg)	97 (7)	112 (8)†	94 (7)	110 (11)†	< 0.001	0.22	0.29
SAP (mmHg)	130 (8)	151 (9)†	127 (9)	148 (12)†	< 0.001	0.16	> 0.99
DAP (mmHg)	79 (6)	90 (7)†	76 (6)	89 (9)†	< 0.001	0.17	0.10
CO (L/min)	6.5 (1.5)	6.8 (1.4)	6.1 (1.0)	6.4 (1.1)	0.06	0.15	0.73
TPR (mmHg/L/min)	15.5 (3.3)	17.5 (3.6)†	15.6 (2.8)	17.9 (3.7)†	< 0.001	0.73	0.31
Foot cold-pressor test (*n* = 9 men)							
HR (beats/min)	66 (7)	69 (9)	63 (8)	66 (9)	0.11	0.06	0.90
MAP (mmHg)	95 (9)	113 (12)†	93 (8)	113 (16)†	< 0.001	0.70	0.19
SAP (mmHg)	130 (10)	153 (13)†	128 (10)	153 (18)†	< 0.001	0.84	0.30
DAP (mmHg)	77 (6)	91 (9)†	74 (6)	89 (12)†	< 0.001	0.34	0.23
CO (L/min)	5.8 (0.8)	6.0 (1.1)	6.1 (1.0)	6.5 (1.1)	0.07	0.17	0.51
TPR (mmHg/L/min)	16.7 (2.4)	19.6 (4.0)†	15.5 (2.6)	17.9 (2.9)†	0.002	0.10	0.54

Values are mean (standard deviation). Data were analyzed with a 2-way repeated-measures ANOVA, followed by Bonferroni post hoc test (*P* < 0.05)

*HR* heart rate, *MAP* mean arterial pressure, *SAP* systolic arterial pressure, *DAP* diastolic arterial pressure, *CO* cardiac output and *TPR* total peripheral resistance

†Significantly different from baseline

## Discussion

The main finding of the present study was that, contrary to our hypothesis, the capacity to upregulate arterial pressure during hand or foot cold-pressor test was not modified after 5 weeks of intermittent exposure to hypergravity. Along with our previous observation that the heart-rate and arterial-pressure responses to an orthostatic provocation also remained unaltered by the specific G training regimen (Eiken et al. 2022), it might, hence, be suggested that, in healthy humans, prolonged iterative gravitoinertial stress within a 5-weeks period does not exert an adaptive influence on the function of sympathetically mediated circulatory reflexes. The failure of developing a sympathetic cardiovascular adaptation may also explain the selective improvement of G tolerance resulted by the G training; the capacity to withstand high G loads was enhanced during the rapid (4 G/s) G-onset exposure, from a mean (range) of 2.9 (2.4–3.6) to 3.3 (2.8–3.8) G, but not the gradual (0.1 G/s) G-onset exposures [pre-training tolerance: 4.4 (3.7–5.3) G, post-training tolerance: 4.5 (3.9–5.4) G] (for details, see Eiken et al. 2022). Thus, when the G load is increased in a slow manner, the contribution of the slow acting sympathetic reflexes is critical to the preservation of head-level perfusion pressure, whereas, by contrast, their compensatory effectiveness is limited in response to the instantaneous fall in head-level pressure occurring during rapid G loading (≥ 1 G/s) (Balldin 1986; Eiken et al. 2022).

Apparently, present results dispute the common notion that the cardiovascular reflex sensitivity is increased in individuals repeatedly subjected to high G loads. Supporting evidence for this has, however, been derived from cross-sectional observations (Convertino 2001; Newman et al. 1998) and longitudinal studies (Scott et al. 2013), in which protection against G by increasing arterial pressures (e.g., anti-G straining maneuvers and suit), was employed during the course of the hypergravity exposures, presumably influencing the process of cardiovascular adaptation. Even though, during the present G-training regimens, each high-G exposure was of sufficient duration to activate sympathetic cardiovascular reflexes, it should be considered that the volume of stress imposed determines the mode and magnitude of adaptation. Thus, it remains to be settled whether a sensitization of sympathetic cardiovascular reflexes, possibly contributing to a greater improvement of relaxed G tolerance, may require higher G loads, more frequent G exposures and/or longer periods of G training. For instance, in small quadruped animals (rats), a month of sustained centrifugation at 3 G enhanced baroreceptor responsiveness (Duling 1967).

Notably, the G-training regimen mitigated the cold-induced pain sensation, especially during the hand cold-pressor test. We have previously found that 5 weeks of repeated and pronounced local intravascular pressure loading, elicited nociceptive habituation, manifesting itself both during application of noxious mechanical stimulus (venous overdistension; i.e., “specific” adaptation) and during thermal stimulus (i.e., “transfer” adaptation) (Keramidas et al. 2021). Even though the present G training pressure-loaded the leg vasculature, it did not alleviate the limb pain engendered by venous overdistension (Eiken et al. 2022; Keramidas et al. 2023). It is, therefore, reasonable to assume that the hypoalgesia noted during local cooling (i.e., a non-specific stimulus) was probably attributable to changes in the subjects’ emotional state (e.g., reduced anticipation and/or anxiety; cf. Dodo and Hashimoto 2017; Burgmer et al. 2011), rather than to any G training-related adjustments in peripheral nociceptor sensitivity. Given the direct link between pain perception and pressor response (Wolf and Hardy 1941; Huang et al. 2021), it might be expected that also the arterial-pressure elevation would have been attenuated post-training. Yet, conceivably, the magnitude of pain reduction was not large enough to blunt the pressure increase.

## Conclusions

Present findings demonstrate that, in humans, 5 weeks of repeated gravitoinertial stress does not alter pressure responsiveness to peripheral sympathetic stimulation. Yet, whether iterative G loading may modulate the function of other, probably more G-specific sympathetic circulatory reflexes, such as the carotid baroreflex and the vestibulo-sympathetic reflex, needs to be examined.

## Data Availability

The data that support the findings of this study are available on reasonable request from the corresponding author. The data are not publicly available due to privacy or ethical restrictions.

## Abbreviations

CO: Cardiac output
G: Dimensionless quantity denoting the ratio between the vector sum of gravitational and inertial forces and Earth’s gravity
+ Gz: High-sustained gravitoinertial force field in the head-to-seat direction
HR: Heart rate
DAP: Diastolic arterial pressure
MAP: Mean arterial pressure
SAP: Systolic arterial pressure
TPR: Total peripheral resistance
